# Visual Associative Learning in Restrained Honey Bees with Intact Antennae

**DOI:** 10.1371/journal.pone.0037666

**Published:** 2012-06-06

**Authors:** Scott E. Dobrin, Susan E. Fahrbach

**Affiliations:** 1 Neuroscience Program, Wake Forest University Graduate School of Arts and Sciences, Winston-Salem, North Carolina, United States of America; 2 Department of Biology, Wake Forest University, Winston-Salem, North Carolina, United States of America; Monash University, Australia

## Abstract

A restrained honey bee can be trained to extend its proboscis in response to the pairing of an odor with a sucrose reward, a form of olfactory associative learning referred to as the proboscis extension response (PER). Although the ability of flying honey bees to respond to visual cues is well-established, associative visual learning in restrained honey bees has been challenging to demonstrate. Those few groups that have documented vision-based PER have reported that removing the antennae prior to training is a prerequisite for learning. Here we report, for a simple visual learning task, the first successful performance by restrained honey bees with intact antennae. Honey bee foragers were trained on a differential visual association task by pairing the presentation of a blue light with a sucrose reward and leaving the presentation of a green light unrewarded. A negative correlation was found between age of foragers and their performance in the visual PER task. Using the adaptations to the traditional PER task outlined here, future studies can exploit pharmacological and physiological techniques to explore the neural circuit basis of visual learning in the honey bee.

## Introduction

The proboscis extension response (PER) is an appetitive associative learning (classical conditioning) task commonly used to study olfactory learning and memory in harnessed insects. Honey bees (and other insects, including fruit flies) reflexively extend their proboscis when a sweet solution (the unconditioned stimulus – US) is touched to an antenna. If this touch is paired with an odor (the conditioned stimulus – CS), a honey bee quickly learns the association and subsequently extends its proboscis to the odor alone [1], [2]. A stable long-term memory can form in as few as three pairings of the CS-US. The time between trials (the intertrial interval, or ITI) can impact acquisition and/or retention in learning tasks; shorter ITIs sometimes yield fewer errors, but spaced conditioning (for example, ITIs of 10 min) can yield superior retention when re-testing is conducted several days after initial training [3]. The antennal lobes (ALs) and the mushroom bodies (MBs) have been identified as sites of convergence of the CS-US signals relevant for olfactory association learning in the insect brain [4].

The mushroom bodies are protocerebral structures found in the brains of all insects [5]. Many studies support the importance of the mushroom bodies for olfactory association learning, typically assessed using the PER task [6]. In honey bees (*Apis mellifera*), the volume of the mushroom body neuropils is related to foraging experience [7]: however, the impact of larger mushroom bodies in experienced forager honey bees on learning or other behaviors has not been studied [1]. A clear link between improved function and size of specific brain areas has been shown in many other species, including humans. For example, the regions of the brain associated with movement and balance are enlarged in skilled golfers and basketball players, and taxi drivers, who require in-depth knowledge of a particular locale, show a positive correlation between years on the job and the volume of the posterior hippocampus, a region implicated in spatial memory [8]–[10]. Experienced foragers may be able to perform mushroom body-dependent tasks better than less experienced foragers because of their larger mushroom bodies. It is to test this prediction that we have focused on development of a visual learning task. Experience-dependent growth of the mushroom body calycal neuropil is best documented for the collar, the visual subcompartment of this neuropil; longer foraging experience is associated with increased dendritic complexity of collar Kenyon cells [11]. We reasoned that, to correlate performance in associative learning tasks with changes in the collar region, a PER task with a visual cue as the CS must be used.

The published literature on visual association learning in honey bees was reviewed to determine how best to assay differences in visual learning correlated with foraging experience and Kenyon cell complexity. The simplicity of the PER method, performed in the laboratory under controlled conditions, is appealing, but the capacity of honey bees to respond to visual cues using traditional methods is controversial. The first report of visual PER in honey bees was published by Kuwabara [2]. This investigator reported that honey bees could learn to respond to the presentation of colors with PER only if their wings and antennae had been removed. Hori and colleagues [12]–[13] studied how honey bees respond to presentation of color and perceived motion. They showed that the compound eyes but not the ocelli (secondary light sensing structures located at dorsal midline on the top of the head) were required for visual learning. Removing the honey bee’s antennae, however, was again reported to be a prerequisite for successful conditioning. Letzkus and colleagues [14] presented an image of a yellow rectangle to foragers without antennae to the right eye only, the left eye only, or to both eyes simultaneously. In this study, the antennectomized honey bees were able to associate a visual cue with a reward; further analysis revealed a right-eye bias in the display of this ability. Niggebrügge and colleagues [15] used visual PER to study the ability of honey bees to generalize or discriminate chromatically similar stimuli with and without antennae. Honey bees were conditioned to respond to the presentation of UV, green, blue, and red lights with PER, and removal of the antennae was once again found to be critical for honey bees to learn to respond to the presentation of a color. Mota and colleagues, however, showed that the honey bees with intact antennae could learn to respond differentially to two colors when the colors were paired with an odor [16]. A similar result was reported by Gerber and Smith [17]. Thus, the literature provides conflicting information: if honey bees with intact antennae can learn visual associations when visual cues are paired with odors, why is visual conditioning without odors only successful in honey bees with the antennae removed? It should be noted that this is not a trivial consideration: intact honey bees are preferred subjects for learning assays because PER performance in honey bees improves significantly when the sucrose reward is applied to the antenna rather than the proboscis [18].

In this study, we differentially conditioned honey bee foragers of varying ages using a visual PER task. The primary goal of this study was to dispute the opinion that antennal ablation is necessary for color learning in harnessed honey bees. Previous studies of PER in honey bees used a collar, typically made of duct tape, for restraint in a small tube. Riveros and Gronenberg [19] used a modified restraint consisting of two insect pins that act as a yoke on either side of the neck to improve the performance of bumblebees in an olfactory PER paradigm. We predicted that this less damaging method of restraint, together with use of a shorter ITI (most previously published studies of visual learning in honey bees have used an ITI of 10–20 min), would permit intact honey bee foragers to learn to respond differentially to visual stimuli. Additionally, we tested the effect of the specific stimulus used in the unrewarded trials on performance in a visual learning task.

## Methods

Honey bee collection and experimental design: Honey bees (*Apis mellifera*) were obtained from research apiaries maintained at Wake Forest University (Forsyth County, NC, USA) using standard commercial techniques. Mass marking techniques were used to find and identify individual honey bees of known age and foraging experience. To obtain newly emerged honey bees, brood combs containing pharate adult workers were removed from field colonies and placed in an incubator (Percival Scientific, Inc., Perry, IA, USA) maintained at 33°C, 35–45% relative humidity. To obtain known age foragers, 100–500 honey bees (<12 h post-emergence) were marked individually on the dorsal thorax with a single dot of enamel paint (Testors PLA, Rockford, IL, USA) 17 times over the course of 2 months, using a new color each day. The marked honey bees were returned to a typical colony at the end of each day of painting. The age of returning foragers captured at the hive entrance could then be determined using a color chart. These honey bees were used to compare the performance of foragers of different ages.

To obtain same-age, precocious foragers, 1200–1500 honey bees (<12 h post-emergence) were marked individually on the dorsal thorax with a single dot of enamel paint in a single day. Together with a mated queen, the marked honey bees were used to establish a new single cohort colony (SCC). The colony was left indoors at 30°C, 30–40% relative humidity, for 2 days before being placed in the field with the entrance closed. A robbing screen was placed at the hive entrance to prevent foragers from neighboring colonies from entering the SCC and to facilitate painting and collection of foragers. Two SCCs were established; once in May 2011 and again in June 2011. To obtain honey bees of known foraging experience, the hive entrance was observed for 5–7 h daily beginning on day 7. Using a new color each day, any focal honey bee (i.e. any honey bee marked with a paint dot on the thorax) observed returning to the hive entrance with a load of pollen or nectar was marked with a second color of paint on the abdomen every day for 5 days.

**Figure 1:**
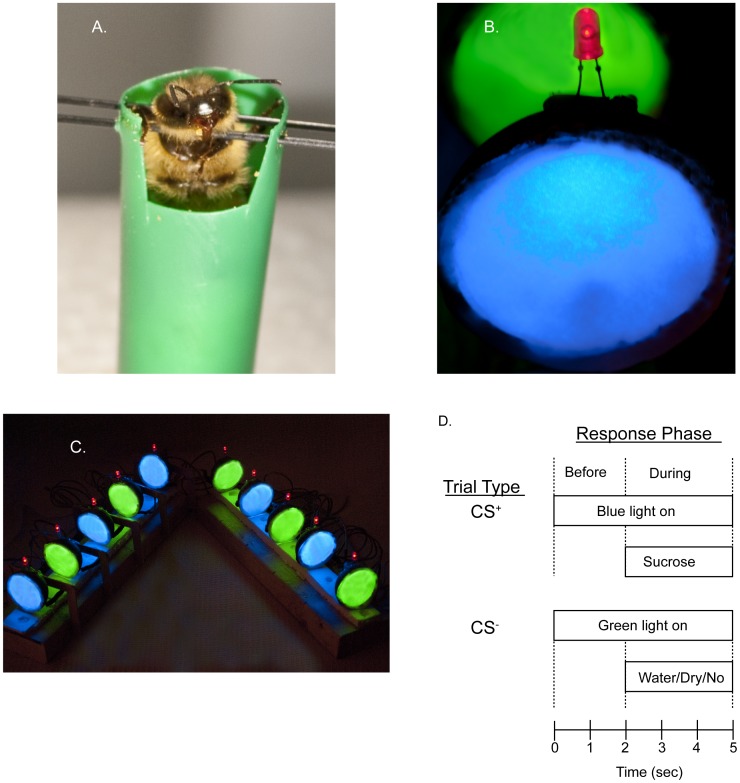
Description of the experimental paradigm. A. Worker honey bees were restrained in plastic drinking straws using a yoke made of insect pins placed on either side on the neck. Honey bees were supported from below using a rolled paper tissue. A small window was cut in the straw to allow full extension of the proboscis. B. Restrained honey bees were placed in front of individual light presentation screens. Each screen could be illuminated with a blue or green led and had a red LED mounted on top to indicate US presentation to the experimenter. C. A series of projection screens allowed simultaneous conditioning of up to ten honey bees. D. Both the rewarded and unrewarded trials used the same timing of CS/US presentation. Following a 3 sec countdown (not depicted), the CS presentation lasted 5 sec during the final 3 sec of which the US was presented. Proboscis extensions (responses) were recorded to the CS before and during the US presentation.

Focal foragers (either normal age or precocious) were collected for use in PER studies by placing a wire screen (3 mm spacing) temporarily over the entrance of the hive to prevent honey bees from entering. For collections from the SCCs, individual honey bees were captured in glass vials and immediately placed on ice in the field. For collections of foragers from the typical colony, batches of 15 honey bees were captured in individual glass vials (each batch taking 10–30 min to collect) before being brought into the laboratory and placed on ice. Once immobilized, honey bees were restrained in individual plastic straws (76 mm×13 mm) with the antennae intact. A small window was cut in the straw to allow the proboscis to freely extend. Rolled tissue paper supported the honey bee from below and 2 insect pins were placed through the walls of the straw, on either side on the honey bee’s “neck” to prevent escape as previously described (Fig 1A; [19]). Honey bees were fed 50% sucrose (w/v) *ad libitum* when they regained movement (approximately 5–10 min after removal from ice) and placed in a dark room (29–32°C) overnight. All subsequent steps were conducted under red light illumination invisible to honey bees [20].

Visual PER conditioning: Fourteen to sixteen hours later, a wooden toothpick soaked in 50% sucrose was touched to the antennae of each restrained honey bee. Only those honey bees that passed this initial screening by performing a prompt PER (a full extension of the proboscis; approximately 25–35% of the total honey bees collected) were included in subsequent conditioning experiments. The identity (i.e., age or foraging experience) of the trained foragers was unknown to the experimenter until after conditioning was completed because the paint mark was not visible once honey bees were restrained. Each of the harnessed honey bees was stationed in front of a projection screen 30–45 min prior to conditioning (honey bees were held in place with clay). The projection screen consisted of a halved racquet ball (5.7 cm or 2.25 in diameter) with a white paper curtain and blue (465 nm±5 nm) and green (525 nm±5 nm) LED lights fixed inside (Fig 1B; Fig S1). The LEDs were chosen because their wavelengths are near the known honey bee photoreceptor maximum sensitivities - S or ultraviolet receptor at k_max_ = 350 nm, M or blue receptor at k_max_ = 440 nm, and L or green receptor; k_max_ = 540 nm [21]. Each LED was aimed to illuminate the inside of the racquet ball directly and indirectly illuminate the back of the paper curtain such that the brightest portion of each LED was aligned. The intensity of each LED was adjusted to 2.96×10^14^ photons/cm^2^/sec using resistors. A red LED (625 nm±10 nm; selected to be undetectable by honey bees) was affixed to the top of the projection screen to indicate to the experimenter the timing of US presentation. LEDs were connected to a U401 USB programmable interface (USBMicro, Mandan, ND, USA) and controlled via custom written software (freely available upon request to S.E. Dobrin). The experimental arena consisted of 10 projection screens, thus allowing 10 honey bees to be tested at a time (Fig 1C). Design plans for the conditioning apparatus are provided in the file Data S1.

Honey bees were trained with 10 rewarded (designated CS^+^) and 10 unrewarded (CS^–^) trials in a pseudorandom order to control for effects of trial order, with an ITI of 5 min. The trial sequence was individually selected for each honey bee via the software. For rewarded trials (CS^+^), a toothpick soaked in 50% sucrose (w/v) was patted on a paper towel to remove excess liquid and touched to the antennae (US^+^), as described [15]–[16]. For unrewarded trials (CS^–^), a dry toothpick or a wet toothpick soaked in deionized water was patted on a paper towel and touched to the antennae (US^−^). If the proboscis was extended in response to the presentation of the toothpick, the toothpick was made accessible to the proboscis and the honey bee was allowed to drink for the remainder of the trial. Each trial lasted 8 sec (Fig 1D). Once the trial was initiated, the experimenter had a 3 sec countdown on the computer screen to identify trial type and prepare accordingly (i.e. hold toothpick near, but out of sight, of the honey bee) before the blue (for CS^+^ trials) or green (for CS^–^ trials) LED illuminated for 5 sec. The red LED illuminated 2 sec later to indicate to the experimenter to present the toothpick to the antennae for the remaining 3 sec of the trial. After noting whether the honey bee responded before and during sucrose presentation, the next honey bee was immediately tested. In pilot experiments, antennae-deprived honey bees performed equally well with either blue or green light in the CS^+^ trials (data not shown).

Experimental groups: For comparison of US^–^ stimuli, the following group codes will be used: *water* (n = 19) refers to the group of honey bees for which a water-soaked toothpick was presented to the antennae during the CS^–^ trials, *dry* (n = 15) refers to the group of honey bees for which a dry toothpick was presented to the antennae during the CS^–^ trials, and *null* (n = 9) refers to the group of honey bees that did not have a stimulus explicitly paired during the CS^–^ trials (in this case, the CS^–^ was the absence of all aspects of the reward, including the touch on the antenna). The number of cumulative responses for each trial was determined and used to classify the trained foragers into learner and non-learner groups. Foragers that responded 3 or more times in the 10 CS^+^ trials were classified as learners; those responding fewer than 3 times out of 10 trials were classified as non-learners. Honey bees that did not extend their proboscis to 3 sequential trials were excluded from analysis. Data obtained from foragers from the SCC and typical colonies were pooled for analysis. Foragers from the SCC were excluded from the analysis of an age effect to prevent complications that may arise from the atypical social status of precocious foragers.

Statistical analysis: The response of each honey bee on a given trial was recorded when the light (CS^+^ or CS^–^) was illuminated (*Before* responding) and during the presentation of the US (*During* responding). As a result, each honey bee had 4 opportunities for a response to be recorded: Before^+^, During^+^ (for responses in the CS^+^ trials), Before^-^, and During^–^ (for responses in the CS^–^ trials). To compare effects of different US^–^ stimuli on learning, a Chi-square analysis was used to compare the number of responses recorded during the final conditioning trial (Prism 5, GraphPad Software, La Jolla, CA, USA). Mann-Whitney U tests, two-tailed Fisher exact probability tests, and Kruskal-Wallis tests with Dunn’s Multiple Comparison *post-hoc analysis* were used, as appropriate, to compare responses to CS^+^ and CS^–^ trials and the number of learners in each group as appropriate (Prism 5). Linear regression was used to analyze trends of performance and age (Prism 5).

## Results

### Intact Foragers can Learn to Respond Differentially to Color Stimuli

**Figure 2:**
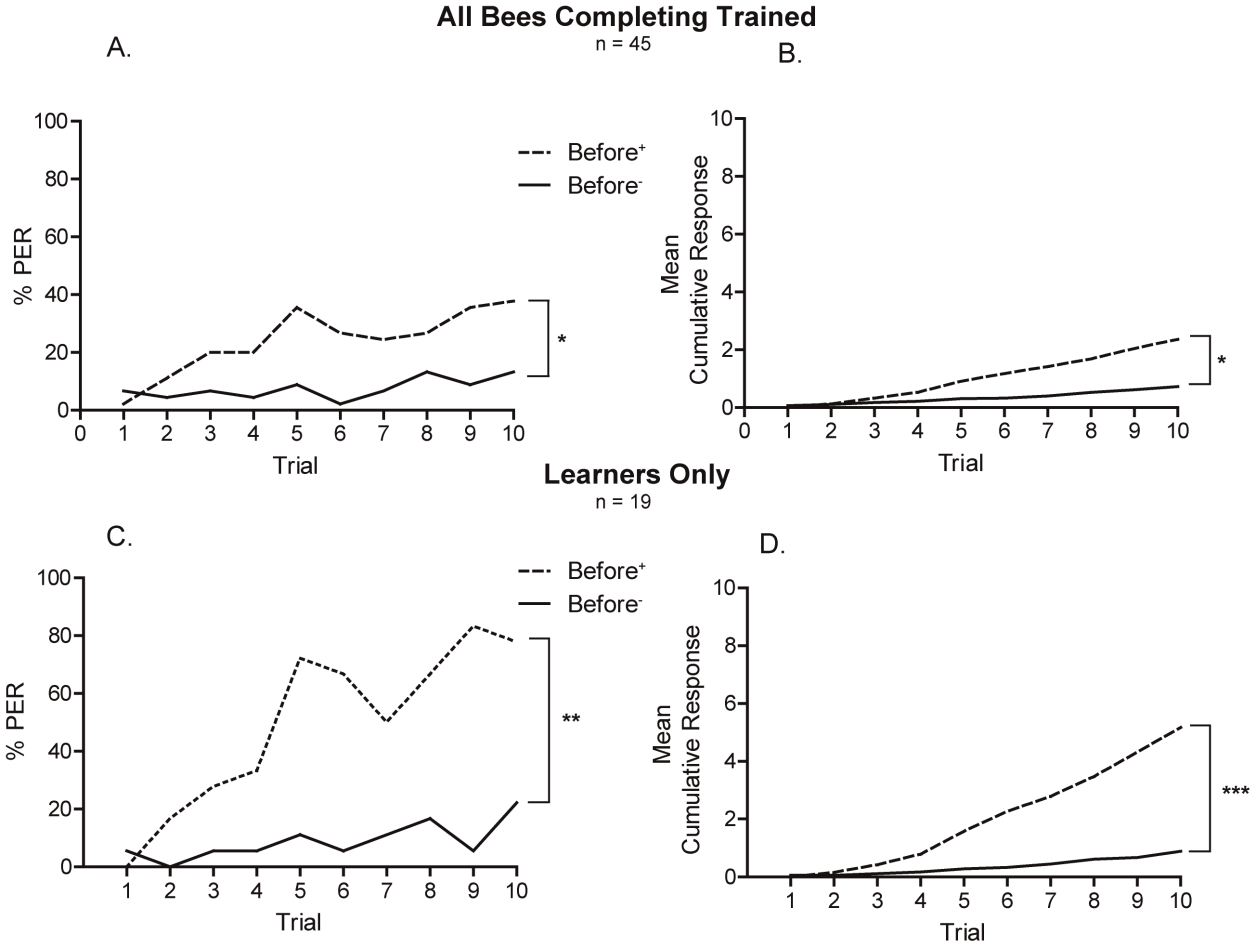
Harnessed, antenna-intact forager honey bees can learn to respond differentially to color stimuli. A, B. All forager honey bees completing visual training. C, D. Only those foragers that responded more than 3 cumulative times to light presentation prior the US (learners). A, C. The percentage of responses to light presentation on each trial. B, D. The average number of cumulative responses. Comparison of responses on trial 10 (A, C) used Chi-square test. Comparison of total cumulative responses (B, D) used Mann-Whitney U test. *p<0.005, **p<0.001, ***p<0.0001.

**Figure 3:**
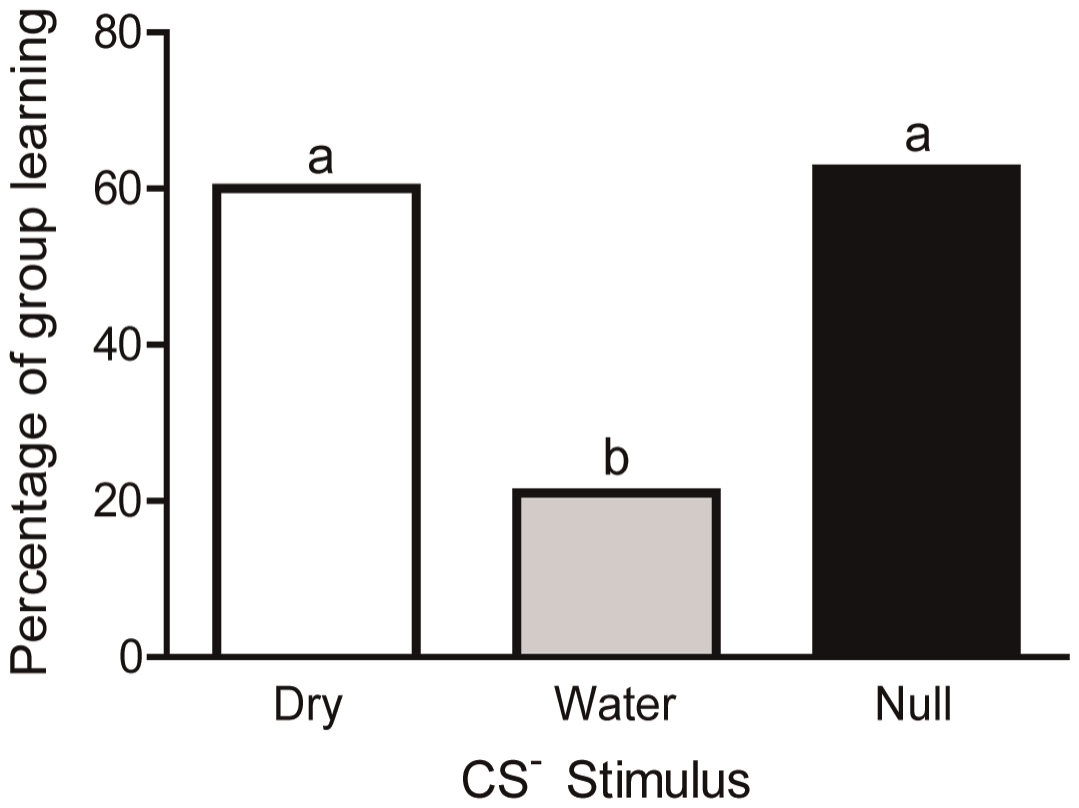
Forager honey bees conditioned with a wet toothpick as the US ^–^ **show reduced learning.** The percentage of honey bees in each group that reached the learning threshold is depicted here. The Fisher exact probability test was used to compare the number of responders in each category (dry: 9/15; water: 4/19; null: 6/9). Letters indicate significant differences (*α*<0.05). Groups designated with the same letter did not differ.

Honey bee foragers were trained on a differential visual association. A blue light (CS^+^) was paired with a sucrose reward and a green light (CS^–^) was paired with no stimulus (*null*) or touching a dry (*dry*) or a water-soaked (*water*) toothpick to the honey bee’s antennae. Overall, foragers responded significantly more frequently on rewarded trials than non-rewarded trials (Fig 2A; χ^2^ (1, N = 45) = 7.5, *p* = 003; Fig 2B; Mann-Whitney U = 652.5, *p* = 0.001). The greatest effect is seen by examining those honey bees that responded in 3 or more trials (“learners”; Fig 2C; χ^2^ (1, N = 19) = 8.5, *p* = 002; Fig 2D; Mann-Whitney U = 44.5, *p*<0.0001). When analyzed separately, both the learners from the *null* and *dry* groups responded differently to the rewarded and unrewarded trials, but no difference in responding to the rewarded and unrewarded trials was found in the *water* group (Fig S2; *dry:* Mann-Whitney U = 0.0, *p* = 0.0003; *null*: Mann-Whitney U = 1.0, *p*<0.018). A significantly lower proportion of honey bees in the water group were classified as learners than those in the dry or null groups (Fig 3; two-tailed Fisher exact probability tests, *p*<0.05; see Figs S2, S3, S4 for further comparison of US groups). No difference was found between the groups of non-learners in their responses to the sucrose presentation on the rewarded trials (see Data S1 for discussion of non-learners), suggesting they all found the sucrose rewarding and remained capable of extending their proboscis throughout the training period. The distribution of the ages of foragers which were categorized as learners and non-learners did not differ (Mann-Whitney U = 92.00, *p* = 0.817).

### Age and Visual Conditioning Performance are Negatively Correlated

**Figure 4:**
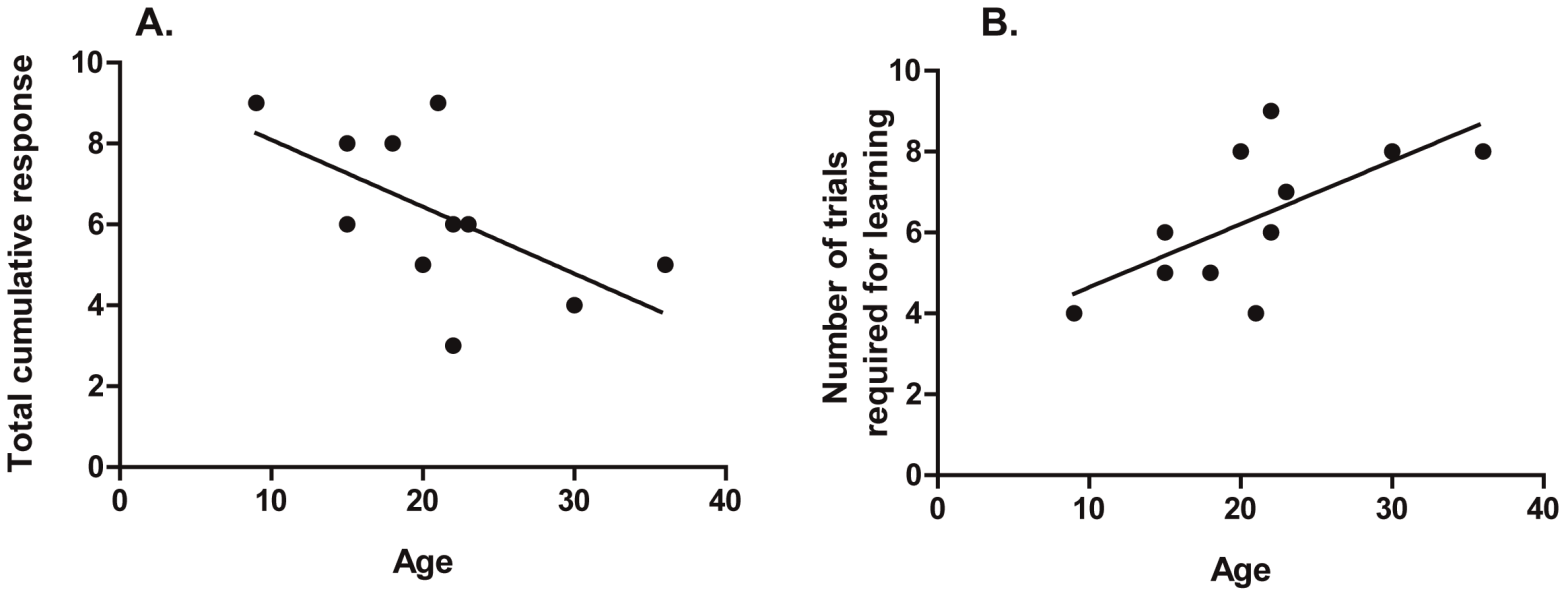
Age of forager honey bees and visual conditioning performance were negatively correlated. A. A negative relationship was found between age of foragers tested in this study and the number of cumulative responses on the rewarded trials prior to sucrose presentation (Pearson’s correlation, r = −0.684, n = 9, *p* = 0.042). B. A positive relationship was found between age and the minimum number of trials required to reach the threshold of learning (3 cumulative responses; Pearson’s correlation, r = 0.719, n = 9, *p* = 0.029).

Honey bee foragers of known age were collected from a typical colony and differentially conditioned. A negative relationship between age and performance was found: younger foragers had a greater number of cumulative responses on the rewarded trials (Before^+^) than older foragers (Fig 4A; Pearson’s correlation, r = −0.684, n = 9, *p* = 0.042). Conversely, a positive relationship was found when measuring the minimum number of trials to reach the threshold of learning (3 cumulative responses; Fig 4B; Pearson’s correlation, r = 0.719, n = 9, *p* = 0.029).

## Discussion

We report here the first example of successful visual PER conditioning in antennae-intact foragers on a differential learning paradigm. Forager honey bees learned to extend their proboscis to the presentation of a blue light after 10 pairings with a sucrose reward. The learners from the *dry* and *null* groups, but not the *water* group, responded differentially to the blue and green lights. A negative relationship was found between age and visual performance on differential training. These results are significant because they contribute to the literature on age- and experience-dependent learning in honey bees and because they demonstrate that visual PER conditioning can be performed in antennae-intact bees. They also suggest that choice of US^-^ can affect the evaluation of learning in the visual PER task.

The main finding of biological significance is that, in this group of honey bee foragers, age and visual conditioning performance were negatively correlated. The link between associative learning and age has been investigated previously using olfactory and tactile PER. Rueppell and colleagues [22] found no correlation with 26–52 day old foragers, an age range that encompasses older forager, within which only two of our sampled honey bees fall. A similar study that also controlled extent of foraging experience found that the older, more experienced bees performed less well than younger, less experienced bees on acquisition of an olfactory PER response [23]. Honey bee pollen foragers can also be conditioned to extend their probosces when a vertical grating is touched to their antennae [24]. Scheiner and Amdam [25] found that more experienced, older foragers showed a greater number of responses to the tactile stimulus 3 days after training than younger, less experienced foragers, but differences were not found 1 or 2 days post-training. Despite the apparent improvement in long term memory reported in the Scheiner and Amdam study, the experienced foragers had lower acquisition curves and were less responsive to sucrose stimulation. These data suggested that, with increasing foraging duration, honey bees have more trouble acquiring new information; but can retain newly-learned information longer. The relationship between foraging duration and learning can now be tested using the visual PER response.

A negative correlation between age and final cumulative response to the CS^+^ was found (fig 4A). Honey bees can initiate foraging as early as 5 days of age, but most begin when approximately 3 weeks old [26]. It is therefore usually reasonable to assume that older honey bees are more experienced foragers than younger honey bees. However, more experienced foragers take foraging flights of longer duration and have higher metabolic demands than younger foragers [27]–[28]. Therefore, it is possible that the most experienced foragers are less likely to survive overnight and continue to respond to sucrose presentation, the requirements for inclusion in this study. If this were the case, the data presented here may represent a covert correlation between visual learning and age of foraging onset. We also did not control for the specialization of the forager (i.e. searching for pollen vs. nectar vs. water), which is correlated with sucrose sensitivity: pollen and water foragers are more sensitive to sucrose than nectar foragers [29]. Scheiner and colleagues compared the performance of nectar foragers on an olfactory PER [18]. Prior to conditioning, the gustatory response score (GRS) of each forager was determined by counting the number of responses to a sequence of increasing concentrations of sucrose. PER performance was positively correlated with GRS. Therefore, it is also possible that foraging specialization or sucrose sensitivity may influence the correlation between visual performance and age we report here.

The main research methods finding reported here is that visual PER conditioning is possible in antenna-intact honey bees. It has been previously reported that it is essential to remove the antennae before training harnessed honey bees on a PER-type visual task [12]–[13], [16]. There are several differences between our protocol and those using honey bees with the antennae removed. The trials were separated by 5 min intervals in this study; most others used 10–20 min ITI [12], [15]–[17]. The previously documented impact of specific ITI durations on acquisition and retention of an olfactory association by harnessed honey bees (as in reference 3) suggests the importance of varying ITI in each new learning task before concluding that learning has not occurred. This feature of our protocol alone, however, cannot explain learning in antennae-intact foragers, as Hori and colleagues [13] used 2 and 5 min ITIs. These investigators nevertheless reported that the removal of the antennae was critical for learning. We suggest that the method of restraint may be an additional critical factor. Previous visual PER studies used a duct tape collar to restrain honey bees in tubes. In this study, we used a yoke made of insect pins placed on either side of the honey bee’s neck to prevent escape. This method was reported to improve performance on an olfactory PER task in bumblebees [19]; method of restraint was also shown to have a significant effect on bumblebee associative learning in a recent study describing the effects of spaced learning on memory consolidation [30]. The aspect of the yoke that is preferred over the tape collar was not identified, but we observed in pilot studies that honey bees that inadvertently had their wings stuck to the tape appeared to be more stressed (e.g. more buzzing and overall activity). We also noted that keeping the honey bees stationary between trials may also have been influential in obtaining successful conditioning. In all studies that explicitly compare honey bees with and without antennae, a single training arena was used and conditioned honey bees were moved into position 30 sec to 5 min before the trial began. In our study, foragers were positioned in front of individual training arenas immediately after the screening step and then not moved until all trials were completed. Creating multiple testing arenas, or creating a projection system that can be easily moved to a stationary subject may be necessary for visual learning. Any or all of these factors likely permitted conditioning of responses by our intact foragers to visual stimuli.

Only one previously published PER study utilized differential conditioning to a visual stimulus. Niggebrügge and colleagues [15] trained honey bees with antennae removed to discriminate chromatically similar stimuli by pairing one color with a sucrose reward and leaving a second color unrewarded. While these authors did not discuss if intact honey bees were tested in preliminary trials, it is possible intact honey bees would not perform as well on this task as they did in the present study.

Differential visual learning may be most successful when the US^–^ is perceived as aversive, in contrast to the rewarding US^+^. Using free-flying foragers in a Y-maze featuring visual cues, Avarguès-Weber and colleagues [31] showed improved ability to discriminate between perceptually similar stimuli when the CS^–^ trial was paired with quinine, a bitter tasting aversive reinforcer. We found that leaving the CS^–^ trial unrewarded (*null* group) resulted in fewer cumulative responses than in the *dry* group (data not shown). One could interpret the difference between the *dry* and *null* groups in our study as the foragers perceiving a dry toothpick to the antennae as more aversive than leaving the trial unrewarded. We also noted that water is not a good choice for use in such studies, possibly because a dehydrated honey bee finds water rewarding.

In summary, the principal finding in this study is that intact honey bee foragers can learn a differential visual learning task. Our data support previous findings that foraging experience is correlated with a deficit in acquisition of an associative memory. Using the modifications to the traditional visual PER outlined here will facilitate future studies that dissect visual learning in restrained honey bees, including any effects on performance resulting from foraging experience. The difficulty of the task can be increased by altering the chromatic differences or complexity of the stimuli to allow a comparison of known age, known experience foragers.

## Supporting Information

Figure S1 **Schematic of visual PER conditioning apparatus.** Up to ten foragers were conditioned in a single training session using two sets of five projection screens and harnesses. A. A lateral view of five harnessed honey bees and the projection screens. B. A forward view of the halved racquetball projection screen (white curtain removed). LEDs of blue, green, and UV (not used in this study) were placed in reflectors inside the racquetball and a red LED was affixed to the top of the racquetball. C. A layout of the one of the ten USB interfaces which interfaced the software with each projection screen.
(TIF)

Figure S2 **Comparison of learners from each US** ^–^ **group.** The responses of foragers to light presentation before US which responded greater than 3 cumulative times (the working definition of a learner in this study) were compared on rewarded and unrewarded trials. Graphs represent the average number of cumulative responses for the *dry* group (A; n = 9), the *null* group (B; n = 6), and the *water* group (C; n = 4). The data represented here are pooled in figure 2A. Statistical analysis of these data used two-tailed paired sample t-tests. *p<0.05, **p<0.01, ***p<0.001.
(TIF)

Figure S3 **US stimuli during the unrewarded trials affected learning.** The responses of foragers which responded more than 3 cumulative times (learners) were compared between different US- groups. Graphs represent the average number of cumulative responses before US on the rewarded trials (A), during US on the rewarded trials (B), before US on the unrewarded trials (C), and during US on the unrewarded trials (D). Letters indicate significant differences as determined by Tukey post hoc analysis (*p*<0.05). Groups assigned the same letter did not differ on that trial. Sample sizes can be found in the legend for figure 2.
(TIF)

Figure S4 **Non-learners responded to sucrose presentation.** The responses of foragers which responded fewer than 3 cumulative times were compared among different US- groups. Graphs represent the average number of cumulative responses before US on the rewarded trials (A), during US on the rewarded trials (B), before US on the unrewarded trials (C), and during US on the unrewarded trials (D). Letters indicate significant differences as determined by Tukey *post hoc* analysis (*p*<0.05). Groups assigned the same letter did not differ on that trial. Sample size for dry = 6, null = 3, and water = 15.
(TIF)

Data S1 **Supplemental results and discussion.** A more complete description of the visual PER conditioning apparatus is included. Additionally, data describing the performance of honey bees using the different US^-^ stimuli and non-learners are included here.
(DOC)
