# Supplementary figures and images for: Visual Associative Learning in Restrained Honey Bees with Intact Antennae
Source: PLoS One. 2012 Jun 6;7(6):e37666. doi: 10.1371/journal.pone.0037666 (PMC3368934; doi:10.1371/journal.pone.0037666)

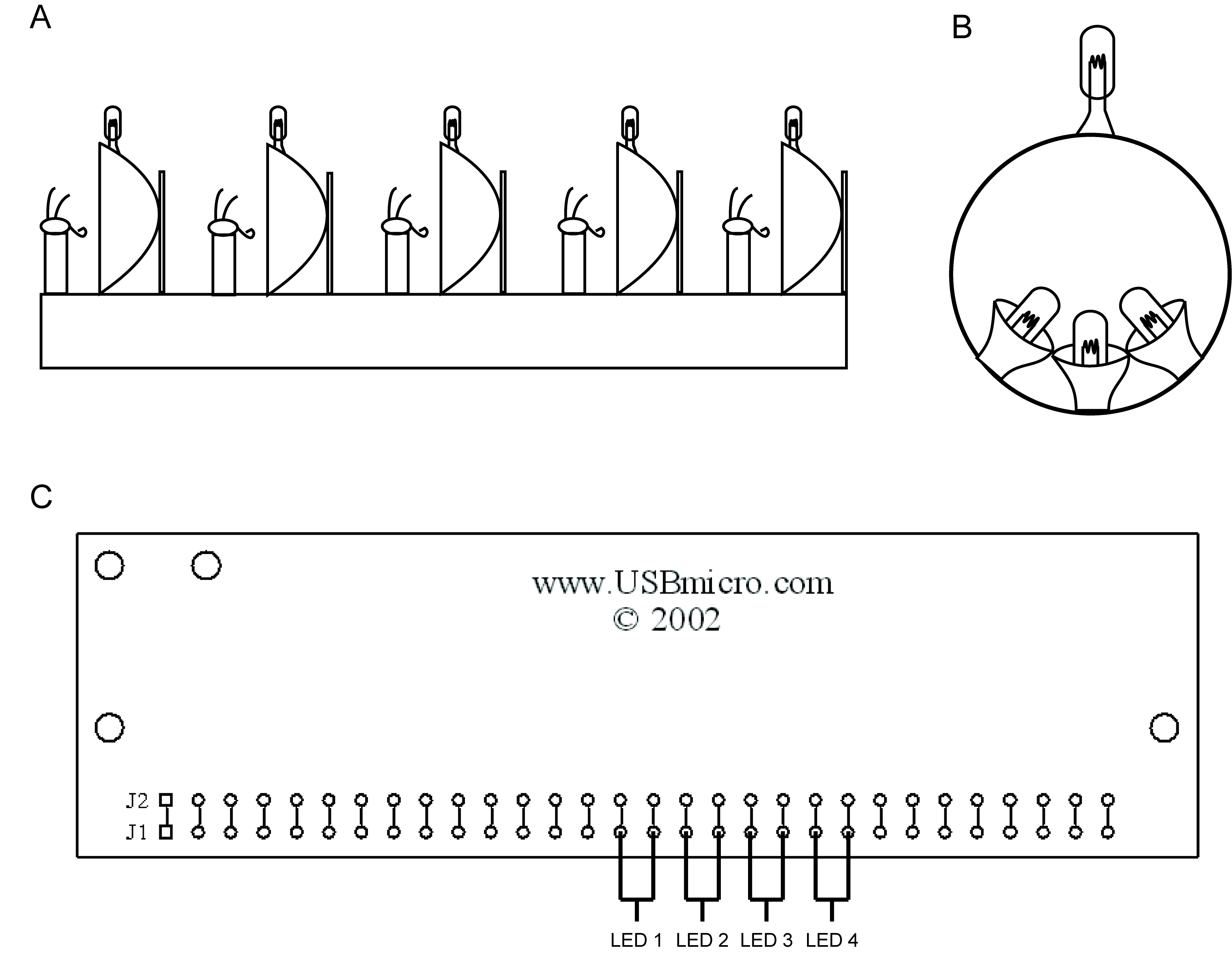

Supplement: Figure S1 — Schematic of visual PER conditioning apparatus. Up to ten foragers were conditioned in a single training session using two sets of five projection screens and harnesses. A. A lateral view of five harnessed honey bees and the projection screens. B. A forward view of the halved racquetball projection screen (white curtain removed). LEDs of blue, green, and UV (not used in this study) were placed in reflectors inside the racquetball and a red LED was affixed to the top of the racquetball. C. A layout of the one of the ten USB interfaces which interfaced the software with each projection screen. (TIF) [file pone.0037666.s001.tif]

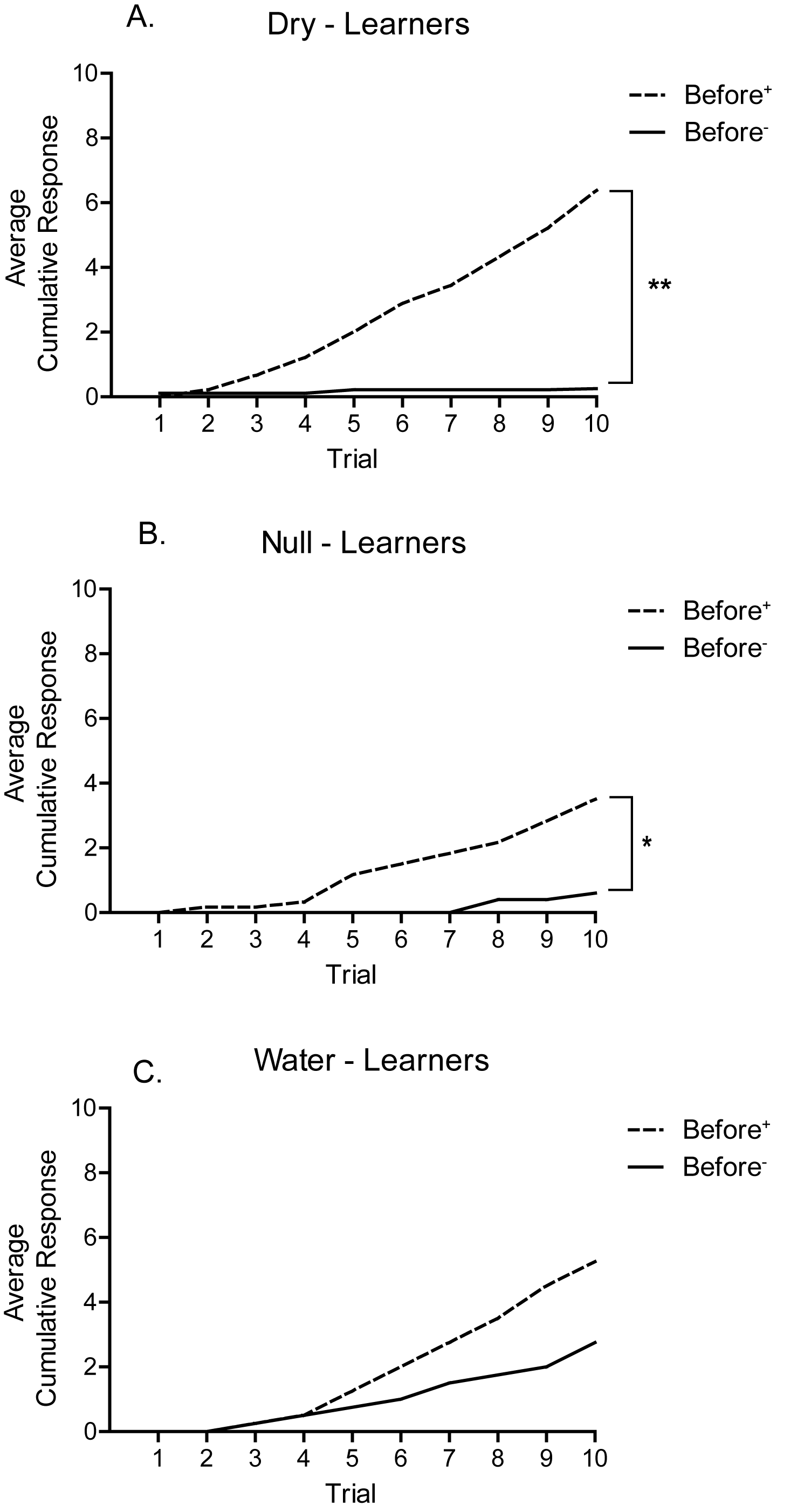

Supplement: Figure S2 — Comparison of learners from each US – group. The responses of foragers to light presentation before US which responded greater than 3 cumulative times (the working definition of a learner in this study) were compared on rewarded and unrewarded trials. Graphs represent the average number of cumulative responses for the dry group (A; n = 9), the null group (B; n = 6), and the water group (C; n = 4). The data represented here are pooled in figure 2A. Statistical analysis of these data used two-tailed paired sample t-tests. *p<0.05, **p<0.01, ***p<0.001. (TIF) [file pone.0037666.s002.tif]

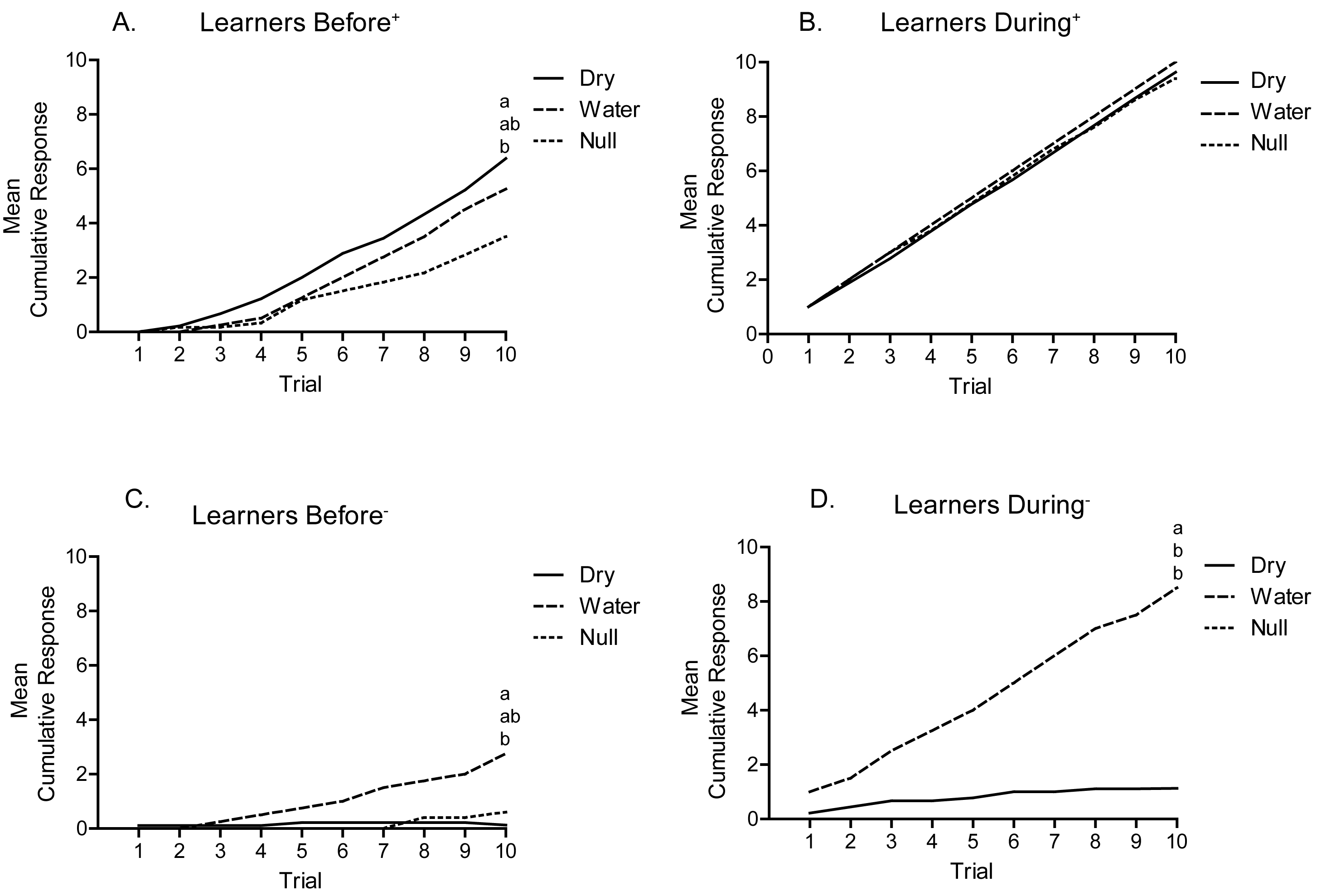

Supplement: Figure S3 — US stimuli during the unrewarded trials affected learning. The responses of foragers which responded more than 3 cumulative times (learners) were compared between different US- groups. Graphs represent the average number of cumulative responses before US on the rewarded trials (A), during US on the rewarded trials (B), before US on the unrewarded trials (C), and during US on the unrewarded trials (D). Letters indicate significant differences as determined by Tukey post hoc analysis (p<0.05). Groups assigned the same letter did not differ on that trial. Sample sizes can be found in the legend for figure 2. (TIF) [file pone.0037666.s003.tif]

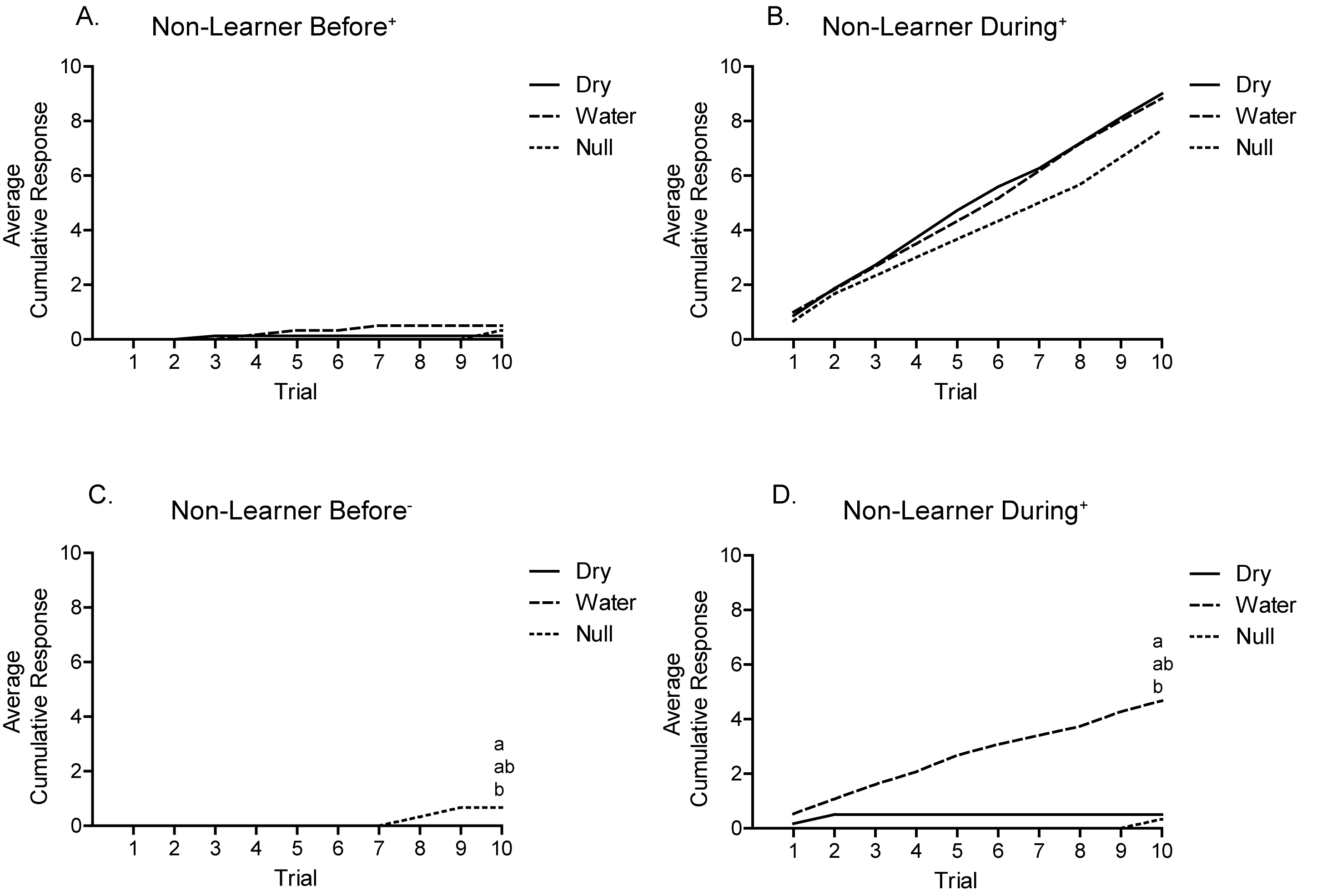

Supplement: Figure S4 — Non-learners responded to sucrose presentation. The responses of foragers which responded fewer than 3 cumulative times were compared among different US- groups. Graphs represent the average number of cumulative responses before US on the rewarded trials (A), during US on the rewarded trials (B), before US on the unrewarded trials (C), and during US on the unrewarded trials (D). Letters indicate significant differences as determined by Tukey post hoc analysis (p<0.05). Groups assigned the same letter did not differ on that trial. Sample size for dry = 6, null = 3, and water = 15. (TIF) [file pone.0037666.s004.tif]
