# Supplementary material for: Visual Associative Learning in Restrained Honey Bees with Intact Antennae
Source: PLoS One. 2012 Jun 6;7(6):e37666. doi: 10.1371/journal.pone.0037666 (PMC3368934; doi:10.1371/journal.pone.0037666)
Supplement: Data S1 — Supplemental results and discussion. A more complete description of the visual PER conditioning apparatus is included. Additionally, data describing the performance of honey bees using the different US- stimuli and non-learners are included here. (DOC) [file pone.0037666.s005.doc]

Visual PER conditioning apparatus

Two identical configurations of five projection screens and harnesses were fastened to blocks of wood (Fig S1A) to allow testing of up to 10 foragers per training session. Individual honey bees were harnessed in drinking straws with a notch removed to allow unencumbered extension of the proboscis and placed 2.5 cm (1 in) from the front of each projection screen. The projection screens each consisted of a halved racquetball with a white paper curtain glued to the cut perimeter of the ball. The inner portion of the ball was painted white and had 3 LED lights with reflectors attached with the electrical leads of the LEDs passing through the ball and sealed (Fig S1B). Each LED (blue: 465 nm ± 5 nm, green: 525 nm ± 5 nm, and UV: 425 nm ± 5 nm (not used in this experiment)) was individually aimed so the brightest portion illuminated the same portion of the rear of the halved racquetball and evenly illuminated the white curtain. A red LED (625 nm ± 10 nm) was affixed to the top of the projection screen to indicate to the experimenter the timing of US presentation. LEDs were connected to a U401 USB programmable interface (Fig S1C) and controlled via custom written software. Software is available upon request.

Visual conditioning differed between US^-^ groups

When the results from the different US^-^ groups were analyzed separately, both the learners from the null and dry groups responded differently to the rewarded and unrewarded trials, but honey bees in the water group did not (Fig S2). No differences were found between groups of learners in their responses to the sucrose presentation on the rewarded trials (During^+^; Fig S3B), however, by trial 8 there were significant differences between the groups in their cumulative responses before sucrose presentation on the rewarded trials (Before^+^; Fig S3A; one-way ANOVA (df = 2, 15), trial 8: *F* = 3.594, *p* = 0.049, trial 9: *F* = 4.522, *p* = 0.029, trial 10: *F* = 4.564, *p* = 0.028). *Post hoc* analysis suggests these reflect differences between the dry and null treatment groups (Tukey *post hoc* analysis, *p* < 0.05 for trials 8 – 10). Honey bees in the water treatment group extended their proboscis significantly greater to the green light (indicating an unrewarded trial) and during the presentation of the CS^-^ than that of foragers in either the dry or null group (Before^-^; Fig S3C, one-way ANOVA (df = 2, 15), trial 7: *F* = 6.737, *p* = 0.008, trial 8: *F* = 6.767, *p* = 0.008, trial 9: *F* = 7.919, *p* = 0.004, trial 10: *F* = 11.911, *p* = 0.001; During^-^; Fig S3D, one-way ANOVA (df = 2, 15), trial 1: *F* = 11.786, *p* = 0.001, trial 2: *F* = 7.500, *p* = 0.006, trial 3: *F* = 15.833, *p* < 0.001, trial 4: *F* = 22.452, *p* < 0.001, trial 5: *F* = 19.313, *p* < 0.001, trial 6: *F* = 23.354, *p* < 0.001, trial 7: *F* = 34.688, *p* < 0.001, trial 8: *F* = 45.559, *p* < 0.001, trial 9: *F* = 46.221, *p* < 0.001, trial 10: *F* = 58.015, *p* < 0.001).

Non-learners are still capable of PER

No difference was found between the groups of non-learners in their responses to the sucrose presentation on the rewarded trials (During^+^; Fig S5B), suggesting they all found the sucrose rewarding and remained capable of extending their proboscis throughout the training period. A significant difference was also found between the non-learners in cumulative responses before and during stimulus presentation in the unrewarded trials (Before^-^; Fig S5C; one-way ANOVA (df = 2, 21), trial 8: *F* = 4.594, *p* = 0.022, trial 9: *F* = 4.594, *p* = 0.022, trial 10: *F* = 4.594, *p* = 0.022; During^-^; Fig S5D; ; one-way ANOVA (df = 2, 21), trial 3: *F* = 5.236, *p* = 0.014, trial 4: *F* = 6.003, *p* = 0.009, trial 5: *F* = 7.289, *p* = 0.004, trial 6: *F* = 7.912, *p* = 0.003, trial 7: *F* = 7.670, *p* = 0.003, trial 8: *F* = 6.990, *p* = 0.005, trial 9: *F* = 7.506, *p* = 0.003, trial 10: *F* = 7.552, *p* = 0.003). *Post hoc* analysis suggests the difference in responses during the US^-^ is because of a significantly greater number of responses to the water than the dry toothpick or not touching the antennae (Tukey *post hoc* analysis, *p* < 0.05 for trials 3 – 10).
